# Abstract of a Lecture on Antidotes to Poison

**Published:** 1846-10

**Authors:** Alfred Baring Garrod


9. *Abstract of a Lecture on Antidotes to Poison.* By ALFRED BARING GARROD, M. D. (Pharm. Jour., from Dub. Medical Press.) The lecturer having defined the term *antidote*, divided antidotes into two classes;—first, those which alter the nature of the poison, and thus prevent its injurious action; and secondly, those which counteract the effect when once produced. The latter class he passed over as being more within the province of the medical practitioner.

The first class, which may be called *direct* antidotes, should be understood by chemists, and their efficacy depends on their immediate administration, which, however, should not supercede the use of the stomach-pump or emetics for the removal of the poison from the stomach.

Dr. Garrod classified poisons into inorganic and organic. Among the former, the mineral acids and the caustic alkalies should be neutralized by substances harmless in themselves and capable of producing inert or inocuous compounds, a circumstance which should always be considered in the selection of an antidote. For instance, solution of caustic potash should not be given as antidote for oil of vitriol, but chalk, magnesia, or bicarbonate of potash or soda. For oxalic acid, lime water or chalk should be given, not potash or soda. For the caustic alkalies, such acids as are harmless should be selected; for instance, citric or tartaric acid, or vinegar, with mucilaginous drinks. For sulphuret of potassium, the best antidote is chloride of soda.

As an antidote for iodine, starch is efficacious when given in sufficient quantity, as it produces an insoluble and inert compound.

Arsenic being a poison more frequently used than any other many antidotes have been tried, among which are sulphuretted hydrogen, sulphur, lime water, &c., but this class of substances are less efficacious than the hydrated peroxide of iron, which, however, must be given in such quantities that there shall not be less than eight or ten grains for every grain of arsenic. Unless given in excess it is not likely to succeed, and emetics or the stomach-pump should also be employed.

Among the mercurial poisons, corrosive sublimate is the most dangerous, and the best antidotes are albumen, gluten, and the proto-sulphuret of iron. Eggs and flour being generally at hand should be given in considerable quantities. The proto-sulphuret of iron is prepared by adding hydrosulphuret of ammonia to a solution of protosulphate of iron. When this is within reach it is useful, as it forms an inert compound of mercury when added to corrosive sublimate.

For the salts of antimony—decoction of oak, elm, or other bark, containing tannin, has been recommended, as this forms an insoluble tannate of antimony.

The salts of lead and baryta may be counteracted by sulphate of soda or magnesia. Albumen and caseine form insoluble compounds with salts of lead; milk may therefore be given. No sure antidote is known for the salts of copper. Sugar has been recommended as an agent capable of reducing copper salts, but it requires for this purpose a higher temperature than that of the stomach. Albumen *in excess* has been proposed; but albuminate of copper is soluble if excess of the sulphate be present. Common salt is the antidote for the nitrate and other salts of silver, as it forms an insoluble chloride.

The next class—namely, organic poisons, are much more numerous than those above mentioned; comprising prussic acid, the alkaloids, (strychnia, morphia, &c.,) as well as all other vegetable and animal poisons. On this part of the subject the lecturer dwelt more at length, and detailed the result of a course of experiments which has led him to recommend animal charcoal as an antidote for vegetable poisons in general, as well as for some of the mineral poisons. It has long been known that charcoal (either animal or vegetable) exerts a peculiar action on colouring matters and absorbs gasses, on which account it is used in filters for purifying water. It has also been observed to throw down certain substances from their solutions, for instance, lime, iodine, &c., as stated by Professor Graham in his Elements of Chemistry. Mr. Warington lately read a paper, at a meeting of the Chemical Society, on the removal of the bitter principle from infusions by animal charcoal. Makers of morphia and other alkaloids are aware that the product is diminished if much charcoal be used. Bertrand tried wood charcoal as an antidote for arsenious acid, corrosive sublimate, and the salts of copper; but in quantities so small as to be inert.

The lecturer had recently introduced the use of animal charcoal as an antidote for some of the inorganic poisons, but more especially for the organic. Before commencing his experiments it had occurred to him that the gastric juice might possibly interfere with the absorption of the poison by charcoal; but this he found not to be the case. His first experiments were with strychnia, which he administered to two guinea-pigs, in one case with animal charcoal, in the other without; and also to several rabbits. In all cases those animals which took the poison with a proper quantity of the antidote, were not at all affected, while the others died. But it appeared that to saturate half a grain of strychnia, two drachms of the charcoal were required, and unless given at least in this quantity, it was not efficacious. The same results were observed in the case of dogs and other animals; one-sixteenth of a grain of strychnia was found sufficient to kill a frog, but with a proper quantity of charcoal a quarter of a grain was given without any poisonous effect. From half a grain to one grain was found to be enough to kill a dog in about ten or fifteen minutes; but when animal charcoal was given in the proportion of half an ounce to each grain of strychnia no effect was produced.

Nux vomica, which in doses of twenty or thirty grains will kill a dog of average size, was rendered inocuous by the administration of half an ounce of animal charcoal.

In the experiments with the pure alkaloids the antidote was given with the poison; in the case of the more mild vegetable poisons, the antidote was administered ten or fifteen minutes afterwards, and mostly with a favorable result.

Similar experiments were tried with opium and its preparations. In giving the tincture of opium it was necessary to take into consideration the effect of the alcohol, two drachms of which are sufficient to kill a dog, and this result is not prevented by charcoal.

The emetic properties of ipecacuanha were found to be counteracted by animal charcoal; and the antidote was found to be equally successful with elaterium, tincture of aconite, aconitine, belladonna, stramonium, hemlock, cantharides, and other vegetable poisons, as well as hydrocyanic acid.

The success of these experiments had induced the lecturer to try the efficacy of animal charcoal as an antidote for the mineral poisons, and with several of them he had found that when given in a large quantity it was more successful than the antidotes usually recommended. In many cases it was found to counteract or lessen the effects of arsenic, corrosive sublimate, the salts of lead and copper; but not so completely as to supercede the necessity of administering such substances as are capable of forming insoluble and inert compounds with the poison.

From the above experiments, the lecturer had come to the following conclusions:—First, that animal charcoal has the power of combining with the poisonous principles of animal and vegetable substances, and forming innocuous compounds: second, that it will absorb and render inert some mineral substances, but, except in the case of arsenic, is not so generally applicable to those poisons as their special antidotes, the quantity required being very great; third, that a certain amount of animal charcoal is required to neutralize the poison —for morphia, strychnia, &c., half an ounce to the grain for the substances from which these alkaloids are obtained, half an ounce to the scruple;—fourth, that the charcoal itself exerts no injurious action on the body.

The kind of charcoal employed in the experiments was the purified animal charcoal, prepared according to the directions of the London Pharmacopæia—namely ivory-black digested in dilute hydrochloric acid, washed and dried. It is improved by heating it to redness in a covered crucible. Ivory-black, if not purified, must be used in much larger quantity, and vegetable charcoal is efficacious only to a comparatively small extent.

In administering the charcoal it should be triturated with lukewarm water, so as to form a fluid of slight consistency, in this way the antidote may be given in ounce doses, or more, according to circumstances. In the selection of emetics, those only should be used which are not rendered inert by charcoal. Ipecacuanha would not operate in contact with it; but sulphate of zinc should be substituted.

In conclusion, the lecturer suggested the propriety of trying animal charcoal as an antidote for other subtle poisons, such as rabies, syphilis, the venom of serpents, &c., applied as a poultice to the part affected, also as a remedy for diabetes, and some other disorders arising from noxious or unhealthy secretions.—*Southern Medical Journal.*

